# Raising the BarCamp: international reflections

**DOI:** 10.15694/mep.2017.000122

**Published:** 2017-07-07

**Authors:** David Topps, Sebastian Dennerlein, Tamsin Treasure-Jones

**Affiliations:** 1 University of Calgary; 2 Graz University of Technology; 3 Leeds Institute of Medical Education

**Keywords:** barcamp, unconference

## Abstract

This article was migrated. The article was marked as recommended.

There is increasing interest in Barcamps and Unconferences as an educational approach during traditional medical education conferences. Our group has now accumulated extensive experience in these formats over a number of years in different educational venues. We present a summary of observations and lessons learned about what works and what doesn’t.

## Background

Across many conferences, there is increasing interest in including Barcamps, Unconferences and various other open session formats that centre upon user-generated topics and discussion. ( Kirstin Duffin, 2012) This originally arose in software development conferences and seemed to be somewhat based on Agile programming concepts, a significant departure from the previous waterfall development approaches where topics were driven top-down in a linear process with no deviation allowed.

There is some debate amongst proponents of these various user-generated formats about the “proper way to do things”, which in itself seems contradictory to the largely organic, emergent structures and processes being promulgated. Barcamps ( https://en.wikipedia.org/wiki/BarCamp) and Unconferences ( https://en.wikipedia.org/wiki/Unconference), as described by Wikipedia (a reference source that is shunned by the more traditional but which seems apt here), have their differences but all of these formats share these main aims and features, which is also backed/ confirmed by more traditional references. ( Budd et al., 2015; Dennerlein et al., 2013; Dennerlein, Gutounig, Kaiser, Barreiros, & Rauter, 2015)

## Aims

• Free knowledge exchange - allow for an open, voluntary, self-motivated, informal and democratic way of communication that encourages every participant to contribute actively to the event.
• Put the audience in the focus - provide greater opportunity for open discussion and knowledge creation than in a traditional conference (presentation and questions) format
• Stay up to date - allow the inclusion of up-to-date and timely topics and work (that may not have been ready for submission months before the conference)
• Co-Create - support emergent learning, providing space for the topic and focus of discussion to change based on the participants’ interests and ideas generated during the event itself

## Features

• No pre-defined sessions or peer-reviewed submission system: participant driven topic generation (mostly on the spot)
• No pre-defined session format: participant driven process (ranging from a presentation over a workshop to a discussion) -- advocated by some experts but others may prefer to have at least some structure.
• No difference between speakers and audience: emphasis on free flow of ideas between participants, in contrast to an experts-novices interaction
• Not outcome oriented: free ranging reporting process

There are other styles of participant-driven discussion, such as Birds-of-a-Feather ( https://en.wikipedia.org/wiki/Birds_of_a_feather_(computing)), PechaKucha ( https://en.wikipedia.org/wiki/PechaKucha), Speed Geeking ( https://en.wikipedia.org/wiki/Speed_geeking), World Cafe ( https://en.wikipedia.org/wiki/World_Caf%C3%A9), which could also be included in this descriptive comparison but we have focused on Barcamps and Unconferences because this is area with which our groups are most familiar.

The goal of the underlying paper is to reflect on the application and benefits of open formats such as Barcamps and unconferences in classical conferences as a “valuable way of getting people involved, making connections, getting creative, achieving goals together, and developing a valuable platform for interactive knowledge exchange” (ref Budd). Based on reflections of the international interdisciplinary expert group, tips and recommendations are inferred for effectively implementing an unconference in a scientific conference. The rest of the paper includes a method section summarizing our understanding of Barcamps, the method of analysis and sample, followed by the depiction of the detailed objective, their reflection in the discussion section and conclusions with an outlook.

## Method

We use this heading somewhat tongue in cheek. For clarity of understanding with our readers, we describe how we have used the Barcamp and Unconference formats, but not with the intent of being prescriptive. In starting out with the basic descriptions, we go on to describe, in action research style, how we have adapted and modified our processes and approach according to the contextual needs of each large group meeting.

In a traditional Barcamp, an overall theme may be chosen (ahead of the event) by the organizers, which serves to elicit the interest of the addressed community and determines the group of attendees. As part of the event itself, participants are strongly encouraged to propose a related topic that they are interested in and are willing to facilitate. Facilitators are discouraged from proposing topics as a promotional stance (whether of a commercial product or concept that they wish to extol upon others). Time should be allowed for this topic generation and selection/voting, which essentially drives the audience-focused character of a Barcamp. Group sizes and divisions are not mandated, but the facilities and availabilities of rooms do present some limits. Participants self-select which topic group in which to participate and are free to move amongst groups according to their own learning needs.

An Unconference is similar and some regard it as a less defined superset of the same principles and largely the same process is followed. Proponents of this format generally suggest that it is even less formatted and there is considerable variation in the process: e.g. an Unconference may not even require the definition of an overall theme for the conference. Emphasis is again made on free exchange of ideas between participants. The facilitator is not regarded as a content expert and their main role is to truly facilitate free-ranging discussion, in similar style to the Barcamp.

Formal recording and reporting of the discussions has been discouraged in some circles with the premise that this allows the groups to generate more free-ranging ideas and intercourse. This is one area that our group has been keen to explore: the contrasting challenges of being creative, while yet fostering the continuation of discussion and creativity beyond the Barcamp session itself.

### Participants

We are basing our descriptions and findings on the cumulative experiences of our team in conducting various Barcamp and Unconference events over the past few years:

• AMEE BarCamp 2016
• Canadian Conference on Medical Education 2008 - 2012
• International Conference on Residency Education 2011
• Medbiquitous Annual Conference 2015
• OHMES Symposium 2016, 2017
• PhD Support Barcamp at the iKNOW conference for 2012-2015
• Barcamp Graz for 2012-2016
• International Medical Educators eXchange (IMEX) in Leeds in 2016
• Knowledge Exchange event in Leeds in September 2016

Just as much about the Barcamp format is non-traditional, we have also been non-traditional in our qualitative discussion-based evaluation of the sessions. While we have made field notes in all the sessions, and recorded some of the sessions with the participants in various means (e.g. post-it, flip chart or google docs), we note that recording the content of the discussions is not accepted by all representatives of Barcamps and Unconferences(see above). We have always followed the informed consent process for all of these sessions. No sessions have been recorded without participants’ knowledge. Most of the times the participants engaged in the recording process themselves in a collaborative manner. We also made it clear that the purpose of our recordings and notes were to improve the processes and logistics and disseminate the results to all participants of the sessions.

It has been impractical to gain ethical certification from the wide number of organizations involved. Our group does attest that all procedures are consistent with the ethical constraints of the Helsinki Declaration.( WMA, 1964)

## Objectives

In exploring the Barcamp and Unconference formats, we had a number of intended objectives, based on our early experiences and associated literature. First, we considered the areas where Unconferences are generally considered to be effective: problem solving - the free-ranging format and democratic style is conducive to vigorous idea generation. Traditional conference formats are designed for idea dissemination; in contrast, unconferencing tries to balance “data pull” with “data push”, that is getting information into the group as well as disseminating it. ( Dennerlein et al., 2015) For some, even this somewhat simplistic description is too redolent of information transfer, and that the process is more about allowing time for building/constructing knowledge as a group, rather than just receiving information, in order to facilitate better shared understanding and knowledge construction, with increased exposure to more viewpoints and perspectives on a topic (in comparison to Q&A sessions following a talk, where time is usually more limited).

We have several objectives in writing this paper. The first is to raise awareness of these informal conference formats. The second is to provide some guidance, based on our experiences, of what to consider when setting up such a format within a conference. The third is to highlight and discuss some issues that we have identified with the formats, and in particular to start a discussion about how to support the further development of ideas that are born within a Barcamp or Unconference:

The Barcamp, which will run at the AMEE 2017 conference (August 26th-30th), will give us an opportunity to try out new approaches to sustaining the ideas and networks arising at this event. We welcome comment and discussion on the approaches suggested in this paper, as this will influence the final set of approaches we choose to use at AMEE this year.

## Resulting Discussions

Rather than use the traditional Results & Discussion format, traditionally associated with study reports, we are blending our findings and recommendations in a somewhat demarchist (or democratic anarchist) style ( http://revelationspace.wikia.com/wiki/Demarchists). In addition, we are grateful to Dr Sklar, a former Editor in Academic Medicine, who gave us great advice about writing up innovations. ( DeVilbiss, 2013) To paraphrase: study participants are always satisfied - satisfaction ratings tell you little. Instead, describe the innovation in enough detail that others can reproduce and improve upon your approach. So here are some of the lessons we have learned through our accumulated experience and participant observations.

### Planning the session

• Allow enough time
  • See Appendix 1: Format or sample schedule
  • Actual topic discussion time often needs only 20-25 mins
    • Don’t go shorter than 15 mins
    • Don’t go longer than 40 mins
  • Trying to cram it into an hour, on an uninitiated group, is pushing things too much
• These sessions can be quite energizing:
  • The elicitation process needs to be calm and fair to allow for the low voices being heard; split down the number of people in case this gets to engaged. The voting process, on the contrary, can be implemented quick and dirty by the level of noise/ number if raised hands or making and counting strokes (with some nice background music).
  • A good session for scheduling just after lunch to avoid the postprandial drowsiness effect commonly seen at meetings
• Critical mass
  • Make sure that the session is suitably advertised so as to generate a critical mass of participants.
  • See Appendix 2: Dissemination before the event
  • Generally, we have found that 30-100 people is ideal.
  • You can conduct a session with fewer people, but their energy and commitment levels may need to be higher.
  • Once you have more than ~120, the session itself starts to take on a greater degree of organizational complexity, which the hosting group should take into account. We speculate that Malcolm Gladwell’s comments about effective group size start to take effect here. ( http://www.wikisummaries.org/wiki/The_Tipping_Point)
• Pick a good overall theme that is
  • of general interest and relevance to your anticipated participants
  • broad enough to allow a range of topics
  • not too broad or impractical in its focus
• Clarify with meeting organizers what is needed for room space
  • Acoustics matter more for unconferencing
    • If they are poor, they are too inhibitory to good discussion
  • Theatre style fixed chairs are an obstruction to good discussion
  • Tables are optional
• Clarify roles in hosting group of organizers
  • Process flow and timekeeping - most sessions will need this specific role, which will effectively preclude more than superficial participation in group discussions.
• Choose an appropriate hashtag
  • Agreeing upon and leveraging the same hashtag allows for parallel and subsequent discussions and potentially even further cooperation on the topics identified in the Barcamp; as well, this contributed to the dissemination of results
  • In smaller Barcamps only one hashtag is needed, in bigger ones there can be one hashtag per stream (sub-camp)

### Equipment needed

• Post-It notes, BluTack, flip charts, walls or easels, marker pens
• Projector/laptop for initial talking points
• Good wifi is essential if you wish to facilitate collaborative note-taking and allow for leveraging social media for extended discussions, input and dissemination
• Note taking - optional
  • Clarify the difference between this and small group discussions with expected report-back
  • Software
  • Social Media channels e.g. a Twitter backchannel

### Recording

Consider whether there is an advantage and purpose to capturing the proceedings. Note the earlier point about this being anathema to some unconference experts. Recording can be useful to:

• To document the process
• To provide follow up on ideas arising
  • Hybrid event that survives beyond the F2F meeting
• But recording can be inhibitory - beware of this effect
• Consent issues
• Catchbox ( https://us.getcatchbox.com/)
  • This is a very interesting device we have seen in action at recent unconferences.
  • Its dynamic element really accelerates the contribution of comments to a large group.

### Voting

Consider whether there is an advantage and purpose to augmenting the voting process:

• New apps such as PollEverywhere
  • But this may also slow things down so we advocate cautious/limited use
• We note also that people effectively vote with their feet, and this is encouraged in the free-ranging style of the unconference.

### Setting the stage

At the start of the event, there are a few factors that help to get things rolling:

• A good description of how unconferencing works, along with the “rules of engagement” (see Appendix 3: The Ten Commandments Evocations of Barcamp)
• It would save time if participants familiarized themselves with the Barcamp process beforehand but this cannot be relied upon
  • Notable lesson from our CCME series, with a high rate of ‘recidivism’ year to year - after the first year, it really took off because the group did not need to be introduced to the process
• Make the short three tag introduction upfront and do not play on this one;
  • i.e. not allow for explanation, no bloating up, but just name and three words
  • 3tag intro allows to understand the audience in the room, adapt the session proposals and get some inspiration for own or shared session proposals
  • Depending on the number of people 5-15min; if the group of participants becomes too big it needs to be split up into the available rooms/ streams (subcamps)

Topic generation

This is a crucial activity in an unconference. Topic areas that are changing rapidly, ideas that are timely and up-to-date (e.g. by allowing wider discussion around topics that have been raised in keynote talks or other sessions,or by allowing the discussion of work that is still in progress or developed after the submission date of the conference), can more easily be accommodated in the unconference format.

Here are a few tips and factors we have noted over the years:

• Sometimes seems time-consuming but worth spending time on
• Everyone does propose sessions (we had feared that some would not want to), but bear in mind this is probably because people who were less keen to contribute decided not to turn up or left quickly (we did see one person leave soon after the rules about participation were stated)
• You don’t own the topic (see Rule #3):
  • one effective approach taken at some unconferences is to suggest that you are free to propose a topic, just because you think it might be interesting to someone, but you don’t own the topic, are not expected to have expertise in the topic, are not expected to facilitate or even to participate in that topic group, if you find something else more interesting.
• No tears and fights over the voting and session choices!
• Topic merging
  • ask proposers to describe their topic (in <20 secs) and agree to merge with another similar topic, if you have too many.

### General Observations

What have we found that the unconference approach can do that other formats cannot?

**Figure 1.**
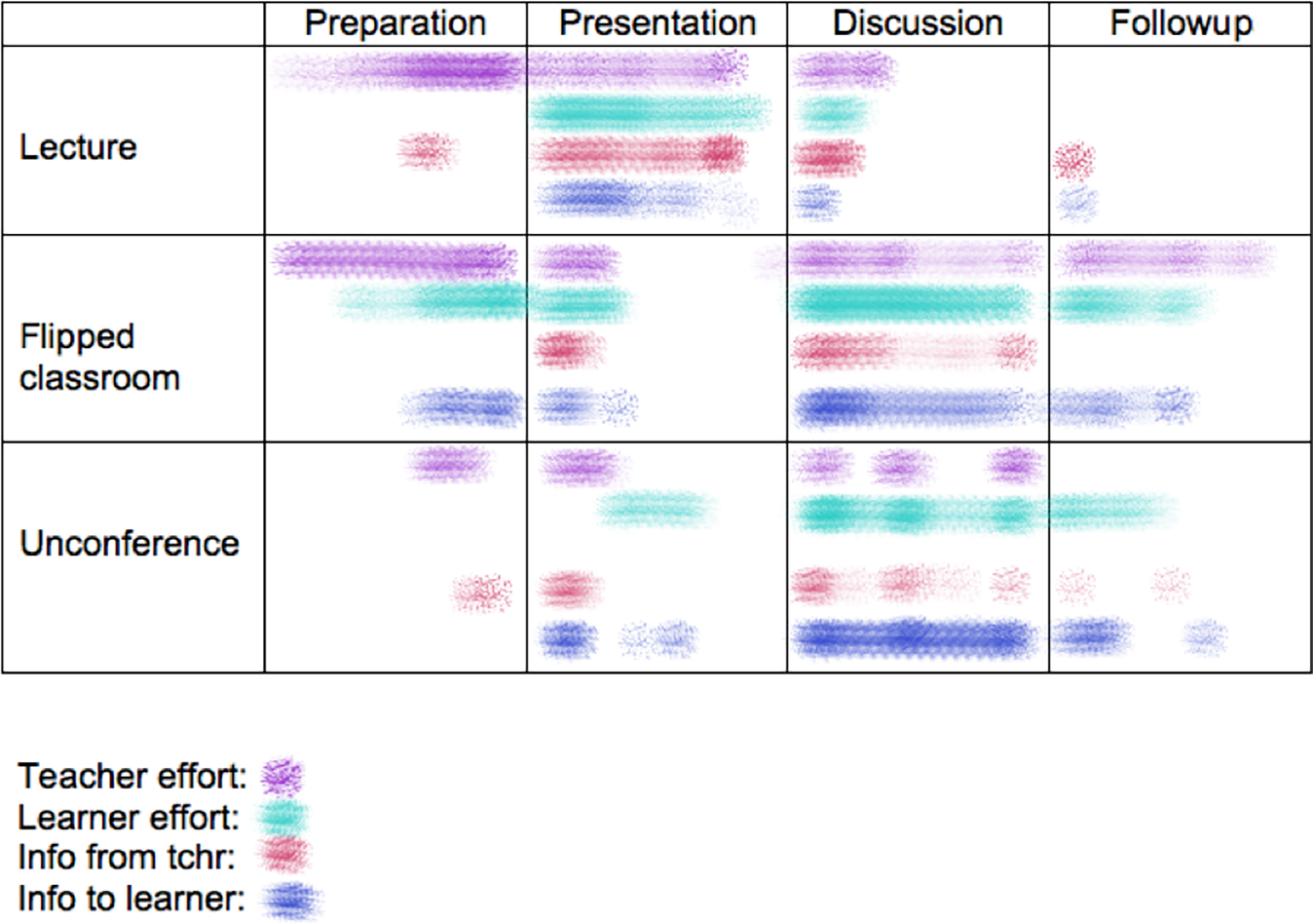
heatmap-timelines showing information economics of learning formats

• Support emergent learning in teams (knowledge co-creation)
  • lets the discussion and interests in the room drive the topic (even changing plans during the session), rather than the schedule determining the topic
  • knowledge creation in terms of teaming up and discussing a topic with an outcome and learnings of all participating members in the discussion/ session
• Compared to standard conference formats (presentations or posters and even workshops) and Pecha Kucha
  • More time and space for discussion
  • More voices/perspectives in the discussion
  • Able to choose topics that are timely and up-to-date
    • that have arisen since the conference submission date
  • Reduces power imbalance between ‘presenters’ and ‘participants’
  • Increased flexibility, can adapt and change sessions even whilst they are running
• Less effortful
  • Consider Figure 1 showing the relative effort or energy expenditure for teacher and learners

Considering Figure 1 which shows the relative effort or energy expenditure for teacher and learners, as illustrated heatmaps along a timeline during the four phases typically found in various learning formats:

• In unconferencing, less presentation prep required.
• But the effort to derive post-event outcomes may be higher -- harder to keep up engagement with the topic discussion group.
• Is this then an analog of the flipped classroom? (Standard classroom: lecture first, homework study afterwards (alone); flipped classroom: study first, generate questions, then group discussion afterwards; standard conference: lecture first, group questions after but limited follow up; flipped (un)conference: no prep or homework, topic/question generation, group discussion, variable follow up).
• You will notice that the effort density and information transfer is maximal during the interactive discussion phase for both the flipped classroom and the unconference.

In terms of impact we have seen how the format itself was picked up and adapted/reused by others in many meetings around the world. There is little doubt that the unconference approach has been found to be highly engaging.

What could be improved?

While the unconference format has been very popular, there are some aspects where there is room for improvement:

• Topic voting

**Figure 2.**
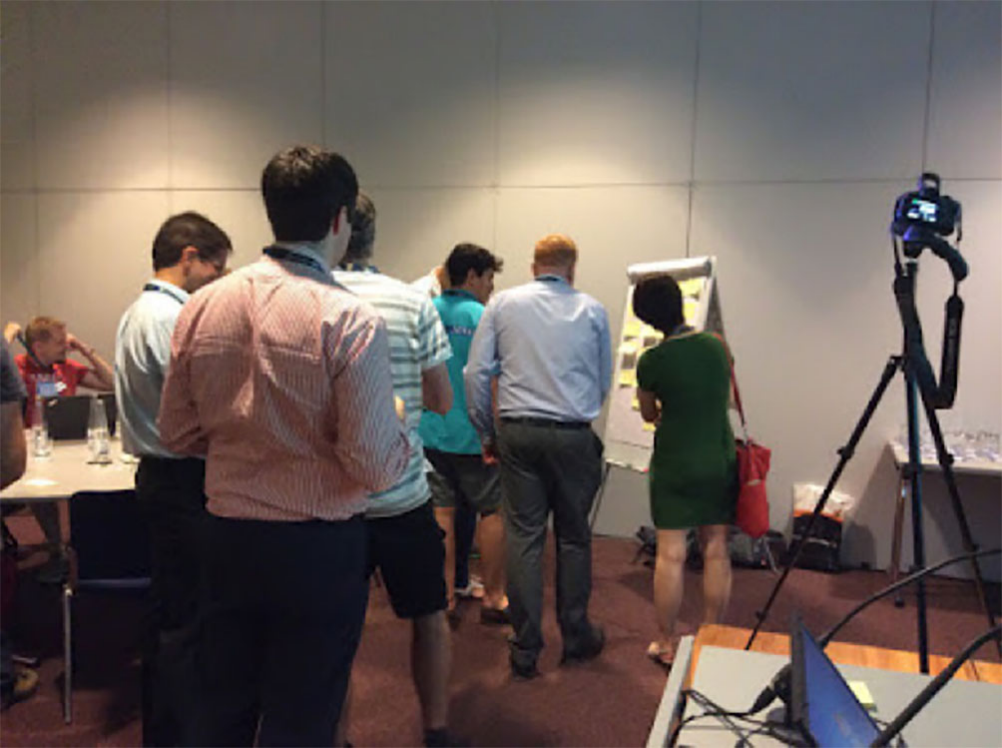
topic voting in progress

• Various attempts have been made to accelerate the topic voting - we have reservations about this.
• Voting process uses up a lot of time - we have spent some effort looking at ways to speed that up this time, such as polling apps like PollEverywhere or Google Forms in combination with our Learning Toolbox. However, some concerns have been expressed that this can inhibit the spontaneity of this dynamic format.
• Collecting session ideas before the event has had limited success with contributions from session facilitators but other registered participants did not send in ideas. It can occasionally be helpful to have a few seed ideas planted amongst likely discussants in order to drive the generation of topic ideas. However, care should be taken not to make this directive, as this can again inhibit the spontaneity of the format.
• But is this lack of prior submissions a problem really? Having a level playing field where all propose sessions at the same time (in the event itself) may actually ensure that all session ideas are treated equally. In contrast if some session ideas have been shared and developed before the BarCamp then (a) they may have a greater chance of being chosen simply due to people’s familiarity with them or their feeling that they are more prepared (b) if they are not chosen then the proposer may feel more disappointed due to the greater time they have invested in the session idea.
• We have participated in some groups who have been quite directive about topic selection, largely pushing the various groups towards preselected topics. We generally counsel against this approach as being inhibitory to good idea generation and wide ranging discussions.

• Idea generation - this is clearly a strength, but do the ideas survive?
  • The unconference format has been criticised for being too ephemeral so what strategies could be employed to improve this?
  • Perhaps there should be more of an online component to the approach.
    • Augmented discussions and collaborative annotation has been tried by our group at some sessions.
    • We have used live editing in Google Docs, and the Learning Toolbox from the Learning Layers project for such purposes.

**Figure 3.**
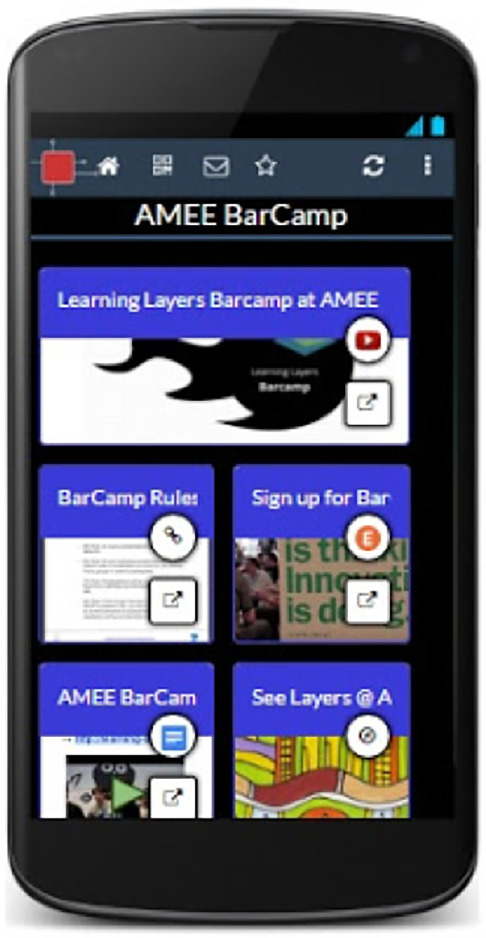
the Learning Toolbox stack from the Learning Layers project

• This does have the advantage of persistence beyond the event and near-ubiquitous access for participants.
• We found these tools were effective at rapidly creating a summary of key discussion points and led to a fairly complete and shared record very soon after the event
• However, we have not yet observed much activity in post-event discussion formats.
  • This may well be a critical mass issue, as is commonly seen in other discussion forums.( Curran & Abidi, 2006)
  • It has not been clear if the ideas discussed in the BarCamp are further developed or adopted by any of the groups.
  • How could we track this more effectively? Social media tracking?
• For some unconference devotees, this represents too much formalization of the process and is regarded as inhibitory. Each group should consider what the main purpose is and whether such augmentation would be productive or counterproductive.

• Support of networking
  • The unconference format generates excellent real-time networking effects through bringing together people with similar interests
    • But only some unconferences have seen persistent collaboration post-event
    • Contact sheets and other sharing mechanisms to allow participants to communicate more easily post-event may be worth exploring.
  • Other mechanisms to improve follow up actions
    • It is good to build upon the energy that is generated.
    • Some evidence with CCME group that building over several years upon existing relationships and shared problem solving continued after each event.
    • This is much less effective with a disparate group.
• Recognition of contributions
  • It is harder to get recognition/publication of ideas and contributions generated using this format. This may have a significant effect on whether participants continue to contribute post-event.
  • Basically, the achieved results are a group product, which are only reachable by the collaboration of all individual contributors. Therefore, each member ‘owns’ the result of a Barcamp.
  • New, more flexible and innovative mechanisms for dissemination of ideas and innovations (such as MedEdPublish, with its post-publication peer-review process) may improve this hitherto barrier.
  • More promotions committees will now consider such contributions as scholarly activities
  • The openness of this approach fits with the current trend towards Open Educational Repositories (OERs)
• Today I Learned (TIL) reporting
  • To try and summarise the proceedings, we used to have group participants make a simple statement at the end of the unconference about what they liked or disliked. However, we found that this often devolved down to the trivial.
  • When we changed the format so as to preface each statement with “Today I learned..”, this was much more effective at reinforcing key learning points from each participant. This might seem obvious but it was dramatic how effective this tiny change was.
  • It also appears to have a synergistic effect, if concordant nodding is a good indicator of agreement and reinforced learning.

## Conclusions

Where next?

This group has been very active in Barcamps and unconferences around the world. We are keen to improve this process and address some of the format’s shortcomings. Here are some areas that we will be working on.

• Iterative process - more to find this year → continuing refinement
  • But remain flexible, context-sensitive, and needs driven.
  • Resist the temptation to nail down your format, in pursuing this refinement
• Improved followup and post-event activity
  • How could we track this more effectively? Social media tracking? Nudges to participants?
  • This is an area we intend to explore further at the AMEE Barcamp in 2017.
• Explore online augmentations further
  • Polling apps - to generate and vote on topics
  • Collaborative note sharing - doing more with Google Docs and Learning Layers
  • Simple project management - Trello etc
• Activity metrics
  • Business world closely follows the outcomes and activities resulting from conferences and trade shows, using Big Data and a wide variety of data sources. In the academic world, we have tended to eschew such data as mere correlations. ( https://xkcd.com/552/)
  • Businesses now die if they do not make good use of big data analytics. Perhaps it is time to come down from the Ivory Tower and learn something from their more innovative stance.
  • It is technically simple to incorporate xAPI reporting ( Haag, 2015) to a Learning Records Store into most of the software applications we now use in education and assessment. Just as clinical medicine is now closely tied to the near-ubiquitous use of electronic medical record (EMR) systems, increasingly in meetings and education sessions, we now make use of many software tools such as virtual learning environments (VLEs/LMSs), collaborative note-taking tools, social media (Twitter, ResearchGate). The Medbiquitous Learning Experience Working Group ( https://www.medbiq.org/learning_experience) is collaborating with many organizations to drive common standards in xAPI related activity reporting.( Topps, Meiselman, & Strothers, 2016)

**Figure:**
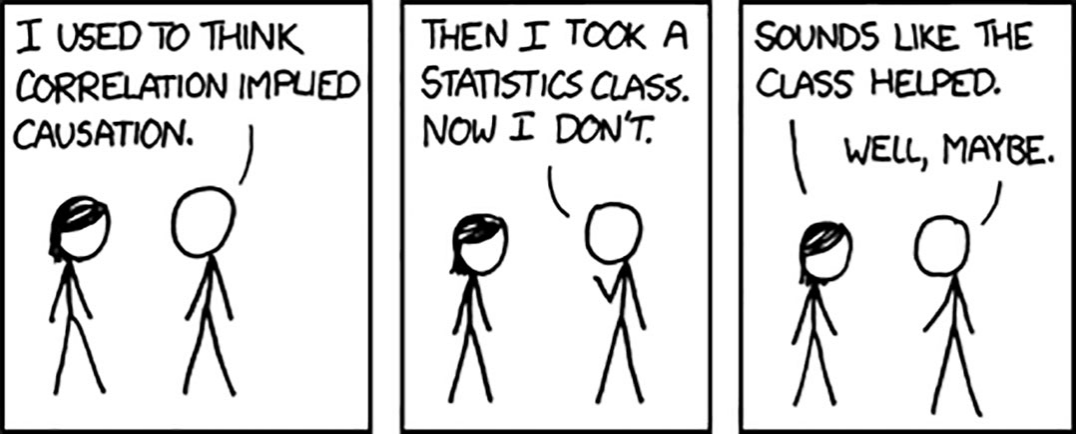


We are interested in having others collaborate in such explorations.

## Take Home Messages

Barcamps and Unconferences provide a new engaging approach that facilitates vigorous group discussions on user-driven topics. It is, as yet, unclear as to what mid to long term impact they have on learning.

## Notes On Contributors

David Topps is Medical Director of the Office of Health & Medical Education Scholarship (OHMES) at the University of Calgary.

Sebastian Dennerlein specializes in new educational methods and technologies at the Graz University of Technology

Tamsin Treasure-Jones is Project Coordinator for the Learning Layers project, at Leeds Institute of Medical Education

## Appendices

### Appendix 1. Format or sample schedule

Here is a suggested schedule for a 90-minute timeslot

**Table:**

Format / Schedule sample	
15 minutes	Introduction to the format • Intro to BarCamp format • 3 tag introductions by participants (optional)
15 minutes	Participants propose and vote on sessions
25 minutes	Session → 4 parallel sessions
25 minutes	Session → 4 parallel sessions
10 minutes	Summary and feedback - Roundup - Round circle “Today I learned.....”

### Appendix 2. Dissemination before the event

**Table:**

Dissemination approaches and materials
• A short video describing the Barcamp format: https://www.youtube.com/watch?v=U5Bkbs7uM3Q • A Learning Toolbox Stack for the BarCamp ( https://api.ltb.io/show/CMKKE) • Tweeting links to the stack, video, EventBrite and the BarCamp Rules

### Appendix 3. The Ten Commandments Evocations of Barcamp

• You do talk about BarCamp (including social media)
• Be ready with a Three-Tag Tell to introduce yourself to the group
• No tourists: be engaged. Be very engaged!
• Propose a topic you are interested in that you think might interest others
• You don’t own the topic, are not expected to have expertise in the topic, are not expected to facilitate or even to participate in that topic group
• If too many topics are proposed, look for a topic you can merge yours with.
• If a topic group is not holding your interest, it is not rude to move; it is rude to stay
• Groups are encouraged to take and share notes to facilitate post-event collaboration.
• There will be no report-back of group discussions by the group facilitator or recorder.
• At the end, each participant will summarize with a “Today I learned..” sentence (not paragraph).
