# Supplementary material for: Raising the BarCamp: international reflections
Source: MedEdPublish (2016). 2017 Jul 7;6:122. [Version 1] doi: 10.15694/mep.2017.000122 (PMC10885275; doi:10.15694/mep.2017.000122)
Supplement: Peer review history

# Reviewer report: Reviewer response for version 1

**DOI:** 10.21956/mep.19313.r28178

**Published:** 2019-04-06

**Authors:** Barbara Jennings

**Affiliations:** 1 Norwich Medical School

This review has been migrated. The reviewer awarded 4 stars out of 5 This paper documents the authors’ experiences of and reflections on the use of BarCamps. It introduces the educational rationale for the BarCamp and Unconference; and offers thought-provoking tips, concrete ideas, and resource links about how to deliver them. I paused to read several of the cited papers and webpages and wondered if there might be a place for speed-geeking and the use of catch-boxes in my own lectures and workshops.I attended the 2016 AMEE BarCamp discussed by the authors, and I found this paper as refreshing and stimulating as I found that event in Barcelona.In their appraisal and reflections of their approach, the authors note that the ephemeral nature of such events can be viewed as a drawback by participants. However, in these metrics-driven times (dominated by structure and time allocation) I view the ephemeral, and opportunity for intangible open-discussion as positives.I have some suggestions to improve this or an updated paper describing the BarCamp method:1. Overall, it could be more concise. There is some duplication of concepts and with a clear link to the documents in the appendix there could be fewer bullet points elsewhere.2. In contrast to point 1, more elaboration is needed to explain concepts that readers will not be familiar with, e.g. in the "setting the stage" section.3. A redrafting could also improve the punctuation and syntax in a few places e.g. in the aims/features sections, for the sake of clarity and precision. 4. What is the source of your data presented in figure 1? Could it be presented more clearly?5. The wealth of linked resources is good in some ways but I wonder if it could now be curated more tightly with more experience and reflection?I recommend this paper to all educators interested in facilitation methods. For those designing and delivering curricula there are some excellent lessons presented about how to engage learners in knowledge construction and discussion, even in large-group settings. This paper has wit. It challenged my assumptions and made me think – that has to be a good thing for any academic.

Reviewer Expertise:

NA

No decision status is available

# Reviewer report: Reviewer response for version 1

**DOI:** 10.21956/mep.19313.r28179

**Published:** 2018-06-18

**Authors:** Anthony David.M

**Affiliations:** 1 Soho

This review has been migrated. The reviewer awarded 4 stars out of 5 I must confess that I had not heard about Barcamps and unconferences till I read this interesting paper. So kudos authors you achieved one of your objectives: raising awareness!A very interesting development indeed. I think such formats will encourage the mavericks and the geniuses to excel. Innovation and creativity get the importance they deserve in such formats. The ten evocations of Barcamp presented are well engineered!

Reviewer Expertise:

NA

No decision status is available

# Reviewer report: Reviewer response for version 1

**DOI:** 10.21956/mep.19313.r28177

**Published:** 2017-07-14

**Authors:** John Dent

**Affiliations:** 1 AMEE

This review has been migrated. The reviewer awarded 4 stars out of 5 This interesting paper describes an alternative approach to the usual conference format which may be unfamiliar to many readers. This report of several such events highlights advantages and challenges and seeks to :- raise awareness of these informal conference formats - provide guidance on how to implement such an event - highlight and discuss some issues that we have been identified The novel approaches to conference meetings reported are relevant to our social-media orientated generation.

Reviewer Expertise:

NA

No decision status is available
